# Jaw in a day: Osseointegration of the implants in the patient’s leg before reconstructive surgery of a maxilla with ameloblastoma. A 4-year follow-up case report

**DOI:** 10.4317/jced.57823

**Published:** 2021-01-01

**Authors:** Pablo Garrido-Martínez, Juan-Francisco Peña-Cardelles, José-Juan Pozo-Kreilinger, Germán Esparza-Gómez, Néstor Montesdeoca-García, José-Luis Cebrián-Carretero

**Affiliations:** 1 DDS, MsC, phD. Associate Professor, Department of Prosthesis, Faculty of Dentistry, University Alfonso X el Sabio, Madrid. Department of Oral and Maxillofacial Surgery, Hospital La Luz, Madrid; 2 DDS, MsC. Professor of the Postgraduate Program in Oral Surgery and Implantology. Universidad Rey Juan Carlos, Madrid, Spain; 3 MD, DDS, phD. Associate Professor of Medicine. Department of Pathology. Universidad Autónoma de Madrid, Madrid. Hospital Universitario La Paz, Madrid; 4 MD, DDS, phD. Professor Titular, Faculty of Odontology, Universidad Complutense de Madrid, Madrid; 5 DMD, phD. Chief, Department of Oral and Maxillofacial Surgery, Hospital La Luz, Madrid; 6 DMD, DDS, phD. Chief, Department of Oral and Maxillofacial Surgery, Hospital La Luz, Madrid Chief of Section, Department of Oral and Maxillofacial Surgery, Hospital Universitario La Paz, Madrid

## Abstract

**Background:**

To describe a clinical case of a cancer patient who underwent ablative tumor surgery, including treatment planning, surgical resection and subsequent implant rehabilitation.

**Case Report:**

A 61-year-old patient with a diagnosis of multicystic follicular ameloblastoma in the maxilla, in which it was necessary a multidisciplinary approach and two surgical steps. In the first surgical intervention osseointegrated implants (OII) were placed in the fibula, until their osseointegration period of 8 weeks was complete. Afterwards, in the second surgery, the micro-vascularized free fibular flap bearing the implants was transplanted into the oral cavity, in order to perform simultaneous reconstruction and early rehabilitation. The final prosthetic rehabilitation consisted in a hybrid prosthesis fabricated using CAD CAM technology.

**Results:**

The latest advances in medical research have improved our understanding of the oral cavity’s regenerative capacity after oncological treatment. This, aided by the advances in digital 3D technologies, has allowed meticulous treatment planning prior surgery.

**Conclusions:**

The functional and esthetic reconstructions described in these two case reports were made possible by coordinating multidisciplinary approaches involving dentists and oral and maxillofacial surgeons. Advances in medicine have improved understanding of the regenerative capacity of the oral region following oncologic treatment, facilitating meticulous advance planning, while advances in digital 3D technologies for planning make it possible to reduce the number of surgical sessions and the time taken for the patient to recover both the esthetics and function of the stomatognathic system.

** Key words:**Oral rehabilitation, oral cancer, oral surgery.

## Introduction

In recent years advances in the diagnosis and treatment of tumors affecting the jaws have made it possible to carry out therapeutic procedures that will restore the patients’ health in cases previously considered untreatable (1). In this context, maxillomandibular reconstruction has always presented a major challenge. In 1982, Taylor described the use of the iliac crest as a flap donor site for mandibular reconstruction, while in 1989 Hidalgo first used the vascularized free fibula flap for mandibular reconstruction following oncologic resection. Since then, use of the latter has become the donor site of choice due to its versatility and the fact that it offers a good length of bone (1,2). To restore function, anatomical reconstruction of the jaw must be accompanied by dental rehabilitation (2).

After oncologic surgery, the patient’s anatomy and occlusal relation will have changed markedly, making patient adaptation to conventional prosthetics complex. In this way, the introduction of osteointegrated implants has represented a revolution in rehabilitation following oncologic treatment of oral and maxillofacial regions as they make it possible to stabilize implant-supported or implant-retained prostheses adequately, and so reestablish the functional capacity of the stomatognathic system. But if functional reconstruction is to be achieved, a key objective is to reestablish the continuity of vascularized bone to receive implants, which will later support prosthetic restorations aimed at occlusal rehabilitation and the restoration of soft tissue sensitivity (3).

Today, guided implant surgery systems represent a major improvement in planning and implant placement in unfavorable anatomical situations (4). In addition, the ongoing evolution of CAD/CAM technologies makes it possible to design and fabricate better fitting structures that will help restore lost function (5).

The aim of this report is to describe a clinical case of a cancer patient, including treatment planning, surgical resection, and subsequent rehabilitation by means of an implant-retained prosthesis.

## Case Report

A female patient was referred to La Paz University Hospital by her dentist to asses a radiopaque lesion in her left maxillary sinus. A biopsy was performed by means of sinonasal endoscopic surgery, revealing a solid multicystic ameloblastoma. Due to the tumor’s benign but aggressive nature, it was removed and rehabilitated employing the “Jaw in a day” technique. Implants were first placed in the fibula until their osseointegration period was completed. Afterwards, the fibula bone was grafted as a free flap into the oral cavity, achieving early rehabilitation.

The patient, a 61-year-old woman, was referred to the hospital’s Oral and Maxillofacial Surgery Unit for evaluation. She presented maxillary edentulism and used a complete removable mucosa-supported denture. The mucosa in the area adjacent to the tumor was intact and free of soft tissue lesions. In the mandible, the lower anterior teeth remained unaltered and she used a removable prosthesis.

**Figure 1:**
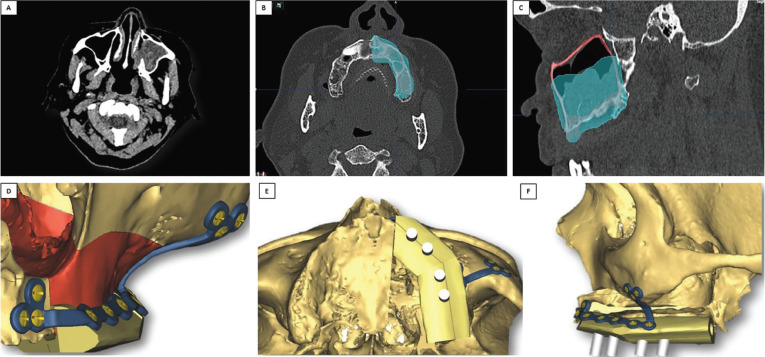
A. Image shows radiopaque lesion occupying the left maxillary sinus. The retained right canine can also be seen. B and C. Cross-sectional CT image shows the entire lesion encompassing the left maxillary sinus, and plan for its removal including safety margins. D, E and F. 3D case planning for placement of a vascularized fibular flap in two sections selected to obtain the shape of the left hemimaxillary arch, bearing four OIIs for subsequent prosthetic oral rehabilitation.

Physical examination did not acknowledge any facial asymmetry or swelling. An axial computerized tomography (CT) scan, showed a lobulated polyp of approximately 3 cm in diameter in the left maxillary sinus (Fig. 1A-C). Considering the clinical characteristics of the lesion, the benign but aggressive tumor required careful treatment planning. As a consequence, the time needed for surgical resection and dental rehabilitation would be reduced, decreasing morbidity as well.

This clinical case involved a multidisciplinary approach and two surgical steps. A technique was used, in which osseointegrated implants (OII) were placed in the fibula, until their osseointegration period of 12 weeks was complete. Afterwards, the micro-vascularized free fibular flap bearing the implants was transplanted into the oral cavity, in order to perform simultaneous reconstruction and early rehabilitation.

To ensure the best possible clinical outcome, virtual planning was carried out using Materialise ProPlan CMF scanner-based image processing software. This allowed a three-dimensional approach of the case, performing virtually the osteotomies in order to place the fibula flap with greater precision. Figure 1 (Fig. 1D-F) displays the treatment plan, with the design of the fibula’s osteotomies, to be used for maxillary reconstruction with optimal OII positioning.

**Figure 2:**
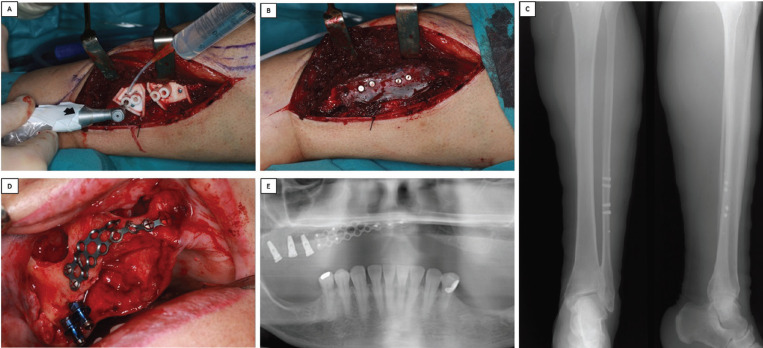
A and B. Photographs show approach to expose fibula bone, placement of four OIIs using a surgical splint and dermis graft of less than 0.5 mm thickness. This was placed in the area of the periosteum of the fibula bone to facilitate subsequent development of periodontal tissue in the reconstructed oral region. C. Radiograph of the fibula, front and lateral projection, showing the four dental implants inserted in the fibula bone base later transferred for oral reconstruction as a vascularized flap. D. Right hemimaxillary approach, with a flap raised to expose the area of the impacted canine and zygomatic apophisis of the maxillary bone, the site of sinus elevation with lateral approach as described by Caldwell-Luc. The image also shows the titanium mesh fixed by osteosynthesis screws. E. Orthopantomograph taken after the first surgical session showing dental implants placed simultaneously to sinus elevation in the first quadrant and extraction of the impacted canine.

In the first surgical intervention a longitudinal incision was performed in the lower left limb to approach the fibula donor site. An osteotomy splint was positioned as anticipated in the virtual planning. The OIIs were placed using a surgical guide following a drilling protocol similar to that used when placing implants in the jaws. In addition, a dermal fat graft with a thickness lower than 0.5 mm was harvested and placed on the bone’s periosteum to provide a basis for future gingival growth (Fig. 2A-C). The procedure continued in the oral cavity, where three OIIs (4 x 10 mm, Zimmer Biomet 3i) were placed in the molar region of the right hemiarch. At the same time, right maxillary sinus elevation was carried out as well as the extraction of a retained canine. Finally, a titanium mesh was placed to reinforce the vestibular cortical area of the surgical site (Fig. 2D,E).

**Figure 3:**
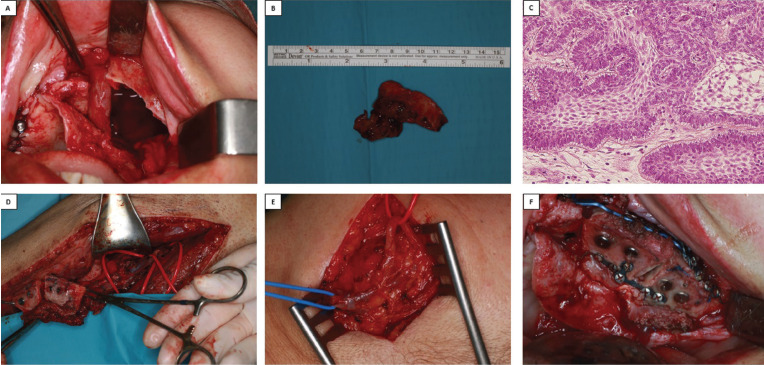
A. Left maxillary defect after excision of the lesion. B. Lesion. C. (x40 enlargement) Images show benign intraosseous tumor composed of odontogenic epithelial proliferation with solid and mulitcystic areas, both cylindrical and cuboid with hyperchromatic nulcei arranged in palisade-like formation, inverse nuclear polarity, and the presence of subnuclear vacuole, which appear similar to dental ameloblasts. In the sub-epithelial area, there are cells of angular outline with lax stroma that exhibit cystic changes reminiscent of the stellate reticulum of a developing tooth. D. Guided, microvascularized fibula flap to be transferred to the oral area under reconstruction, showing the position of the fibular artery. E. Positions of artery and facial vein for subsequent anastomosis with the fibula artery and vein placed in the intraoral region. F. Adaptation of the microvascularized fibula flap at the maxillary defect host site.

The second surgery consisted in the resection of the left maxilla ameloblastoma and its simultaneous microsurgical restoration with a fibula free flap. Under general anesthesia, an intraoral maxillary approach was performed. A crestal incision was made from the first quadrant molar region to the tuberosity of the second quadrant, with a central release. Both the maxilla and the OIIs in the first quadrant were exposed. A fourth OII was then placed in the canine region of the first quadrant, after removing the titanium mesh from the area. A left hemi-maxillectomy was carried out, including the lesion, following the virtual planning previously performed (Fig. 3A). An intra-operative biopsy was done, verifying negative margins (Fig. 3B,C). Afterwards, the fibula flap was harvested (Fig. 3D), while a cutaneous incision was made beneath the left mandibular border to dissect the facial vessels, preserving the marginal branch of the facial nerve (Fig. 3E). The micro-vascularized flap was adapted to the maxillary defect on the left side, placing three preformed titanium plates. Subsequently, the microvascular anastomosis between the peroneal and facial vessels was performed. The cutaneous incision was then sutured, as well as the intraoral approach (Fig. 3F). Finally, a CT scan was carried out to radiologically asses the fibula flap’s position.

**Figure 4:**
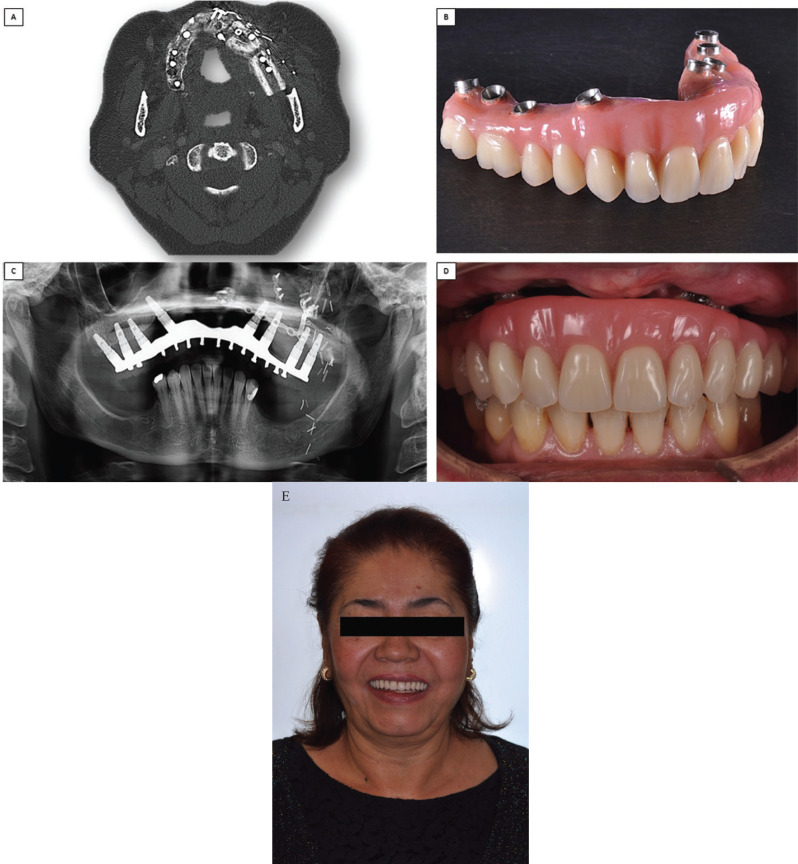
A. Radiological control after surgery. B,C,D,E. Images of definitive prosthesis and its adaptation to the patient’s maxilla after 4 years of the second surgery.

There was a delay of 30 days between surgery and the beginning of prosthetic rehabilitation. This period would allow a decrease of the inflammatory response and adequate tissue healing to ensure the correct registration of the future prosthesis. Due to the loss of volume and function in the upper lip area, the chosen rehabilitation was a metal-resin hybrid prosthesis. Consequently, transepithelial abutments (MultiUnit ®) were placed, in order to aid passive adjustment, as well as to decrease bacterial filtration and peri-implant bone loss. Once adequate occlusion and esthetics were verified, a laser-sintered cobalt chrome screw-retained framework was fabricated using CAD CAM technology. Finally, the structure was covered in resin and Phonares (Ivoclair ®) teeth. Mucosal support was not provided in the surgical site, where no keratinized gingiva was present. Alternatively, enough space was maintained to guarantee proper hygiene and visual clinical follow up (Fig. 4A,B)

After a 4 year follow up, the patient remains free of disease and the implants and prosthesis are in good condition. Figure 4 (Fig. 4C-E) shows the oral status of the prosthesis.

## Discussion

The restoration of normal function after ablative tumor surgery presents major challenges. Oncologic and trauma defects in the jaw, accompanied by ambitious treatment objectives in terms of function and esthetics, augment the complexity of the treatment procedures involved, constituting a research topic that requires exhaustive analysis. The current tendency is toward achieving precise defect reconstruction involving fewer surgical sessions, aimed at restoring structure, function and appearance almost immediately (6).

Reconstruction can only be achieved through advanced microvascular surgery, making it possible to perform vascularized grafts that ensure better bone consolidation, and healing with less risk of infection (7).

The fibula has been shown to be a reliable and adapTable donor site for reconstructing this type of defect, due to its length and the possibility of obtaining a large vascular pedicle. This makes it possible to perform osteotomies and reconstructions that will provide favorable facial contours, as well as an adequate host site for OIIs to support subsequent prosthetic restoration (2,8,9).

The “Jaw in a day” technique has developed over several years, through modifications to the vascularized FFF. It was first reported in 1996 by Vinzenz *et al.*, in two cases of patients who had undergone resections of the jaw to treat tumoral processes, although both cases used the shoulder blade as donor site (10).

Levine *et al*. argues that the technique described in the present report, in which OIIs were placed in the fibula, waiting 3 months for their osteointegration before transferring them as an intraoral free flap to reconstruct mandibular or maxillary bone, has only become possible due to the development of three-dimensional digital technologies for virtual surgical planning, making it possible to generate 3D models from CT scans before surgery (11).

Almost simultaneously to Levine *et al.*, Rohner *et al.* treated four cases, two with malign neoplasias, adopting the same approach, although treatment was carried out following treatment of the tumoral processes by chemotherapy and radiotherapy (12).

Following these four published cases, Schepers *et al.* reported the case of a patient with osteonecrosis in which OIIs were placed in the fibula, and then in a second surgical session, the necrotic tissue was excised and the fibular carrying the OIIs placed to reconstruct the mandible (13). Quasi *et al*. published three more similar cases in which benign tumors were treated (14). After this, Runyan *et al.* reported the technique in a patient who had been previously treated for a malign tumor, in this case a Ewing sarcoma (15).

Yetzer *et al.* published the case of a young woman with refractory sclerosing osteomyelitis in the mandible, with reconstruction and oral rehabilitation using the technique described here (16).

The case by Salman *et al.*, is about of a young man who underwent this technique for rehabilitation of a mandibular defect after excision of a keratocystic odontogenic tumor (17). The latest published case was by Pauchet *et al.*, whose place a three implants in a left mandibular defect, which was not specified the etiology of the defect (18).

The Table 1 shows all the cases published to date using this technique. Most of them were performed to restore mandibular defects, with fewer in the maxilla. The present case describes the application of the technique in maxillary bone. Moreover, most of the cases reported to date treated defects resulting from the removal of benign tumors. The second case reported here followed the excisional biopsy of an ameloblastoma, this being the most common type of tumor removal reconstructed by this technique, with five cases involving ameloblastoma reported in the literature.

**Table 1:**
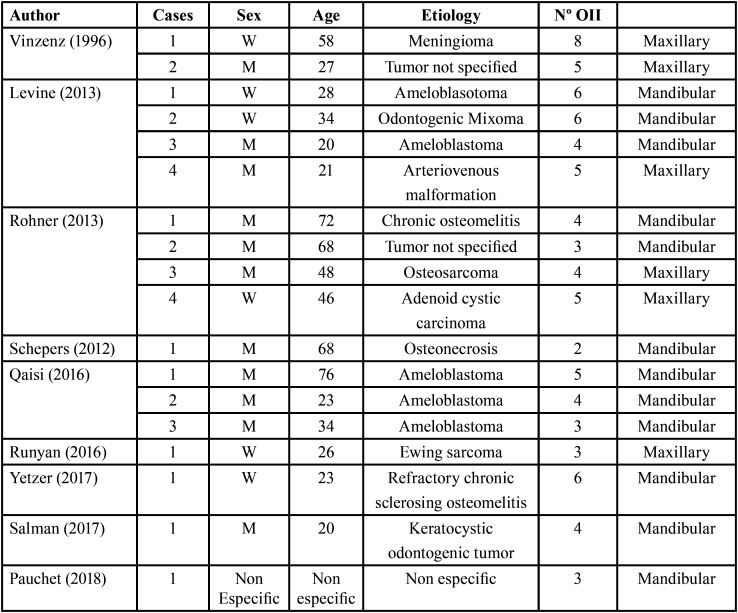
Cases published in the literature, employing the oral rehabilitation technique described in the present case report. Abbreviations: W: Woman, M: Man.

Virtual advance treatment planning for surgery and oral rehabilitation after the tumoral process as described above makes it possible to carry out the various treatment stages correctly and precisely and restore the patient’s oral health.

## Conclusions

The functional and esthetic reconstructions described in these two case reports were made possible by coordinating multidisciplinary approaches involving dentists and oral and maxillofacial surgeons. Advances in medicine have improved understanding of the regenerative capacity of the oral region following oncologic treatment, facilitating meticulous advance planning, while advances in digital 3D technologies for planning make it possible to reduce the number of surgical sessions and the time taken for the patient to recover both the esthetics and function of the stomatognathic system.
